# Squalene Stimulates a Key Innate Immune Cell to Foster Wound Healing and Tissue Repair

**DOI:** 10.1155/2018/9473094

**Published:** 2018-09-30

**Authors:** Cristina Sánchez-Quesada, Alicia López-Biedma, Estefania Toledo, José J. Gaforio

**Affiliations:** ^1^Center for Advanced Studies in Olive Grove and Olive Oils, University of Jaén, Spain; ^2^Immunology Division, Department of Health Sciences, Faculty of Experimental Sciences, University of Jaén, Campus las Lagunillas s/n, 23071 Jaén, Spain; ^3^Agrifood Campus of International Excellence, ceiA3, Spain; ^4^University of Navarra, Department of Preventive Medicine and Public Health, School of Medicine, Pamplona, Spain; ^5^Centro de Investigación Biomédica en Red Área de Fisiopatología de la Obesidad y la Nutrición (CIBEROBN), Madrid, Spain; ^6^IdiSNA, Navarra Institute for Health Research, Pamplona, Spain; ^7^CIBER-ESP. Instituto de Salud Carlos III, C/ Monforte de Lemos 3-5, Pabellón 11, Planta 0, 28029 Madrid, Spain

## Abstract

Anti-inflammatory effects of virgin olive oil (VOO) have been described recently, along with its wound healing effect. One of the main minor compounds found in VOO is squalene (SQ), which also possesses preventive effects against skin damage and anti-inflammatory properties. The inflammatory response is involved in wound healing and manages the whole process by macrophages, among others, as the main innate cells with a critical role in the promotion and resolution of inflammation for tissue repair. Because of that, this work is claimed to describe the role that squalene exerts in the immunomodulation of M1 proinflammatory macrophages, which are the first cells implicate in recent injuries. Pro- and anti-inflammatory cytokines were analysed using TPH1 cell experimental model. SQ induced an increase in the synthesis of anti-inflammatory cytokines, such as IL-10, IL-13, and IL-4, and a decrease in proinflammatory signals, such as TNF-*α* and NF-*κ*B in M1 proinflammatory macrophages. Furthermore, SQ enhanced remodelling and repairing signals (TIMP-2) and recruitment signals of eosinophils and neutrophils, responsible for phagocytosis processes. These results suggest that SQ is able to promote wound healing by driving macrophage response in inflammation. Therefore, squalene could be useful at the resolution stage of wound healing.

## 1. Introduction

Mediterranean countries have been differentiated from others because of their low prevalence of certain inflammatory diseases, such as cancer and cardiovascular diseases [1, 2]. Recently, it has become clear that diet plays a central role in the appearance and development of chronic inflammation, and some studies describe the “inflammatory potential” of certain diets [3]. Olive oil, the main fat of the Mediterranean diet, has been shown to possess anti-inflammatory effects in several diseases [4, 5]. Many of its minor compounds have been shown to prevent or treat several different diseases [4], but there is no description of which olive oil compounds are responsible for these anti-inflammatory properties and whether they can be used in several inflammatory diseases, such as Crohn's disease, inflammatory bowel syndrome, and cancer.

**Figure 1:**
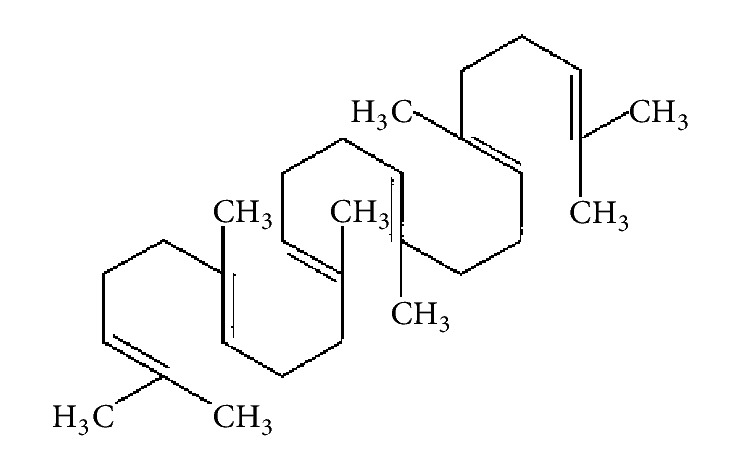
Chemical structure of squalene (SQ).

Consumption of virgin olive oil (VOO) is associated with a low prevalence and incidence of certain diseases [4, 6, 7]. Consequently, VOO compounds have been studied extensively. Squalene (SQ) (2,6,10,15,19,23-hexamethyl-2,6,10,14,18,20-tetracosahexane) (Figure 1) is the major component of the nonsaponificable fraction of VOO. Furthermore, this compound is found in human, animal, plant, and microbial cells as a precursor of sterols and of many other bioactive terpenoids. Its consumption is 10-fold higher in Mediterranean countries than in Northern European countries or the United States due to the intake of VOO [7]. More recently, SQ has been used in several applications, including chemopreventive in several tumours [8, 9].

SQ is the main minor compound found in virgin olive oil (from 0,8 to 13 g/Kg) [10] and has been described to possess preventive properties against cancer [8], skin damage [11], and atherosclerotic lesions [12]. Nevertheless, there have been no studies to describe its role in macrophages, key innate immune cells responsible for development of the wound healing process properly.

Inflammation is a complex set of interactions among soluble factors and cells and can arise in any tissue in response to traumatic, infectious, postischemic, toxic, or autoimmune injury. Macrophages are the main and first cells that appear at the injured area and are capable of managing and controlling the inflammatory response. M1 phenotype macrophages are usually cytotoxic effectors that mediate the Th1 cytotoxic and proinflammatory response, whereas M2 phenotype macrophages possess anti-inflammatory properties and drive the Th2 inflammatory response [13]. Following the interaction of both types of macrophages, the healing process normally leads from infection to recovery. These macrophages are critical to wound healing, and without their presence, the inflammatory phase results in impaired wound closure, granulation tissue formation, severe haemorrhage, and later wound closure in the early stages [14]. Several studies suggest that wound healing begin with a M1 macrophages response that derive in a M2 macrophage phase, where the wound is definitively closed and decontaminated [14].

However, if targeted destruction and assisted repair are not properly phased, inflammation can lead to persistent tissue damage from leukocytes, lymphocytes, and collagen. Inflammation can be considered in terms of its checkpoints, where binary or higher-order signals drive each commitment to escalate, “go signals” trigger “stop signals”, and molecules responsible for mediating the inflammatory response can also suppress inflammation depending on the timing and context. The noninflammatory state does not passively exist because of the absence of inflammatory stimuli; rather, the maintenance of health requires the positive actions of specific gene products to suppress reactions to potentially inflammatory stimuli that do not warrant a full response [15]. Therefore, persistent chronic inflammation could drive several diseases, such as inflammatory bowel syndrome and cancer, and chronic inflammation occurs prior to the appearance and development of cancer [16, 17].

In our study, the effects of squalene (SQ) on the proinflammatory responses of M1 macrophages were studied. The cytokines and molecules involved in wound healing process were studied after SQ treatment in a human monocyte cell line (THP-1), which was differentiated into M1 macrophages. To our knowledge, this is the first time that SQ has been studied in a macrophage cell line experimental model.

## 2. Materials and Methods 

### 2.1. Chemicals

The following were purchased from Sigma-Aldrich Co. (St Louis, MO, USA): squalene (SQ) 2,6,10,15,19,23-hexamethyl-2,6,10,14,18,20-tetracosahexane (squalene CAS 111-02-4) purity ≥98%); hepes solution; sodium pyruvate solution; nonessential amino acids mixture 100× (NEAA); lipopolysaccharides from* Escherichia coli* 055:B5 (LPS); 2,3-bis(2-methoxy-4-nitro-5-sulfophenyl)-2H-tetrazolium-5-carboxanilide inner salt (XTT sodium salt) (purity ≥90%); N-methylphenazonium methyl sulphate (PMS) (purity ≥98%); phorbol 12-myristate 13-acetate (PMA) (purity ≥99%); phosphate buffer saline (PBS); sodium chloride (NaCl) (purity ≥99,5%); L-arginine (L-Arg) (purity 98.5-101.0%) suitable for cell culture and Triton X-100. Foetal bovine serum (FBS) was obtained from PAA Laboratories GmbH (Pasching, Austria). Minimum essential medium with Eagle's salts (MEM) and phenol-red-free Roswell Park Memorial Institute 1640 medium (RPMI) were obtained from Gibco® Life Technologies Ltd. (Paisley, UK). Methanol dry (max 0,005%), magnesium chloride (50% MgCl2 powder QP) (MgCl2), and ethanol absolute PRS were purchased from Panreac Quimica S.L.U. (Barcelona, SPAIN). TrypLE Express was obtained from Invitrogen (Eugene, OR, USA). *β*-Mercaptoethanol was purchased from Applichem GmbH (Darmstadt, GERMANY). PIPES (98,5+%) was obtained from Acrōs Organics (Geel, BELGIUM). Culture plates were obtained from Starlab (Hamburg, GERMANY).NF-*κ*B p65 Sandwich ELISA kit was purchased from Cell Signaling Technology (CST, Danvers, MA, USA). RayBio® Human Cytokine Antibody Array (Human Inflammation Array I) was purchased from RayBiotech, Inc. (Norcross, GA, USA). TNF-*α* Enzyme Immunometric Assay Kit were purchased from (Stressgen) Enzo Life Science, Inc. (Farmingdale, NY,USA).

### 2.2. Cell Culture and Treatment

The THP-1 (human acute monocytic leukaemia) cell line was obtained from American Type Culture Collection (ATCC, Rockville, MD, USA). THP-1 monocytes were maintained at 37°C in a humidified atmosphere under 5% CO2 in MEM supplemented with 10% FBS, 1% hepes buffer, 1% sodium pyruvate, 1% NEAA, and 0.05 mM 2-mercaptoethanol. THP-1 cells were subcultured at least twice per week and discarded and replaced with frozen stocks after 25 passages to achieve optimal growth conditions.

Macrophage differentiation was induced by treating THP-1 cells (1x10^6^ cells/ mL) for 24 h with 50 nM of PMA, followed by a period of further culture without PMA. PMA-differentiated THP-1 cells (1.5 x 10^5^ cells/mL) were stimulated for 24 h with LPS (1 *μ*g/mL) to induce M1 macrophages and was followed by squalene (SQ) treatments at 1, 10, and 100 *μ*M for 24 h [5]. All of the assays were conducted under these conditions, except for those specified below.

### 2.3. Cytotoxicity Assay

THP-1 cell survival, measured as the cellular growth of treated cells versus untreated controls, was conducted using an XTT-based assay according to our previous publication [5]. PMA-differentiated THP-1 cells stimulated after 24h with LPS (M1 profile macrophages) were used in this assay. Briefly, cells were seeded into 96-well culture plates in a total volume of 100 *μ*L per well. After overnight incubation to allow cell attachment, 100 *μ*L of fresh medium was added, which contained increasing concentrations of SQ from 3.12 *μ*M to 100 *μ*M, and incubated for another 24 h. Thereafter, cells were incubated with XTT in Phenol-Red free RPMI medium for 3 h, and the absorbance was measured at 450 nm wavelength (620 nm as reference) using a plate reader (TECAN GENios Plus, Tecan Trading AG, Switzerland). Viability was calculated using the formula

(1) $$\begin{array}{c} \%\;\;viable\;\;cells=\left[\;\frac{\left(A\;\;treated\;\;cells\right)}{\left(A\;\;control\right)}\;\right]\times 100 \end{array}$$

where A is the difference in absorbance between optical density units (A = OD_450_ – OD_620_). All measurements were performed in quadruplicate and each experiment was repeated at least three times.

### 2.4. RayBio® Human Cytokine Antibody Array in M1 State THP-1 Macrophages

Differentiated THP-1 cells were stimulated with LPS (1 *μ*g/mL) for 24 h. After that, the cells were treated with SQ. Subsequently, the supernatants were isolated and processed according to the manufacturer's instructions. Array membranes were directly detected using a chemiluminescence imaging system (FluorChem E System, ProteinSimple) to detect the production levels of the cytokines/proteins represented in Table 1.

Data were analysed with the RayBio® Human Inflammation Antibody Array 1 Analysis Tool (Cat # SO2-AAH-INF-1). Data are expressed as chemiluminescent arbitrary units acquired by the chemiluminescence imaging system (FluorChem E, Protein Simple, CA, USA) after normalization (positive control) and background subtraction.

### 2.5. TNF*α* Production

After obtaining the results from the cytokine array, TNF*α* molecules were measured to corroborate the production of anti-inflammatory cytokines in M1 macrophages. After treatment with SQ for 24 h, TNF*α* production was measured with the TNF-*α* Enzyme Immunometric Assay Kit (Stressgen) according to the manufacturer's protocol using a microplate reader (TECAN GENios Plus, Tecan Trading AG, Switzerland). Data are expressed as the mean (of three independent assays) of the total produced protein (pg/mL).

### 2.6. NF-*κ*B Detection in M1 State THP-1 Macrophages

After stimulation of differentiated THP-1 cells with LPS (1 *μ*g/mL) and SQ treatments, NF-*κ*B production was measured according to the manufacturer's protocol (PathScan Total NF-*κ*B p65 Sandwich ELISA kit (Cell Signaling Technology)). Cells were analysed using a microplate reader (TECAN GENios Plus, Tecan Trading AG, Switzerland). Data were expressed as the main (of three replicates) with respect to control, which was set at 100%.

### 2.7. NO Production in M1 Type THP-1 Macrophages

Nitric oxide (NO) production was measured according to methods described by F. Amano with some modifications [5]. Differentiated THP-1 cells (5x10^5^ cells/mL) were seeded on a 12-well- plate and treated with SQ at 1, 10 and 100 *μ*M for 24 h. Subsequently, LPS (1 *μ*g/mL) and L-Arginine (L-Arg) at 10 mM were added to the wells and incubated for 24 h. Supernatants were collected and incubated with absolute ethanol for 30 min at -20°C. Later, supernatants were centrifuged at 10,000 xg at 4°C for 10 min and, finally, they were aliquoted. NO production was analysed with a NO analyser (NOA 280i de SIEVERS, GE Water and Process Technologies, Pennsylvania, USA). Data are expressed as the mean of three independent experiments relative to untreated control, which was set to 1.

### 2.8. Statistical Analysis

For all assays, except for the cytokine antibody array, data are displayed as the mean of at least three independent experiments (±SEM) run in triplicate. The results of the cytotoxicity assay are expressed as percentages relative to the untreated control cells (which was defined as 100 %). General variance analysis (ANOVA) and Student's* t*-test were conducted. A p value <0.05 was considered to be statistically significant. These statistical analyses were performed using Statgraphics Centurion XVI statistical software (Statpoint Technologies, Inc., Warranton, VA).

## 3. Results

### 3.1. Effects on Cytotoxicity

THP1 cells that polarized to the M1 phenotype were treated with different concentrations of squalene (SQ). This compound did not show any toxic effects (statistically significant) on the survival of these macrophages (Table 2). The results are shown as percentages of cell survival with respect to the control, which was set to 100%. Any of the treatments were significantly cytotoxic, but the range between 3.12 *μ*L and 25 *μ*L appear to decrease cell survival percentage in 15-20% approximately. On the contrary, at the highest concentration the cell survival appeared to increase.

### 3.2. RayBio® Human Cytokine Antibody Array in M1 State THP-1 Macrophages

**Figure 2:**
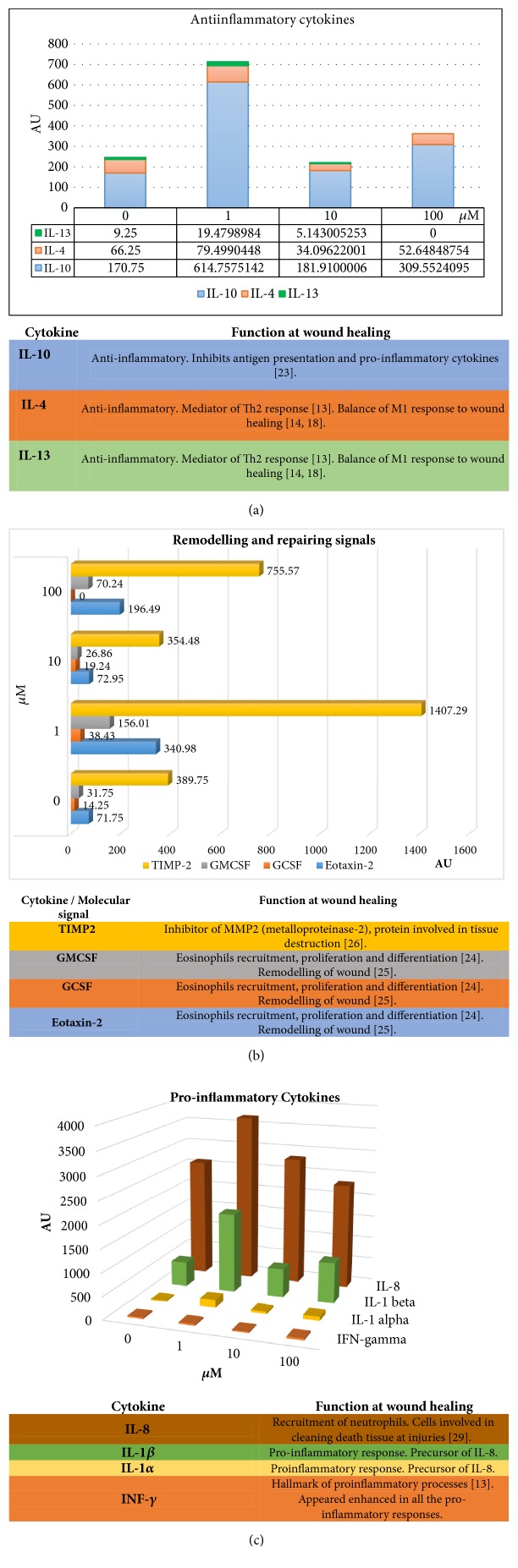
Production of cytokines in M1 macrophage cells after SQ treatment in chemiluminescent arbitrary units (AU). (a) Anti-inflammatory cytokines; (b) molecular signals of remodelling and repairing damage; (c) proinflammatory cytokines. At 1*μ*M the anti-inflammatory and recruitment effect are more notable than the rest of concentrations used. With this concentration SQ appeared to stimulate more efficiently an anti-inflammatory accumulative activity in M1 macrophages.

The higher anti-inflammatory effect was observed after treatment at low concentration (1 *μ*M). IL-10, IL-4, and IL-13 was enhanced after 1 *μ*M SQ treatment in levels that other concentrations tested did not reach. However, this effect was lost when the concentration was increased (Figure 2(a)). According to this SQ appearing to modulate the secretion of these cytokines, anti-inflammatory IL-10 cytokine is dramatically enhanced and IL4, which possesses an anti-inflammatory role. Moreover, interestingly IL-13 is enhanced but inhibited at 100 *μ*M. Both, IL4 and IL-13, are involved in a Th2 response, which could balance the M1 response. Unless SQ at higher concentrations appeared not to promote IL4 and IL-13 compared with control, at 1*μ*M this compound increased dramatically IL-10, IL-4, and IL-13, three of the main anti-inflammatory cytokines synthetized by M2 macrophages [18]; however, a M1 phenotype was induced before SQ treatment on the macrophage.

After treatment with SQ at 1 *μ*M, M1 macrophages showed an increase in production of eotaxin-2, GCSF, GMCSF, and TIMP-2. At higher concentrations, the levels of eotaxin-2, GMCSF, and GCSF did not change, and TIMP-2 showed elevated levels with 100 *μ*M of SQ (Figure 2(b)). All these molecules are involved in tissue remodelling and anti-inflammatory response to infections. All of them conform the response to damage leading by M2 macrophage after tissue injuries. This way, a M2 profile and response are enhanced by SQ at this concentration in macrophages with a M1 profile.

IL-1*α* production was enhanced at all of the concentrations assayed, with an increment at 1 *μ*M of SQ, followed by IL-1*β* and IL-8. IL-1*α* is also enhanced at 1 and 100 *μ*M with respect to control. INF-*γ* production did not show any difference with respect to the control, except at 10 *μ*M. At this concentration, INF-*γ* production was decreased (Figure 2(c)). INF-*γ* is one of the central signals of proinflammation and M1 response. This cytokine recruit more macrophages and polarize them into a M1 profile, which could in turn conserve the proinflammation response. IL-1*β* and IL-8 are proinflammatory signals but can play a restructuring role, promoting neutrophils to phagocyte other apoptotic cells and clean the area. Taking this into account, IL-1*β* and IL-8, unless both are proinflammatory cytokines, could act as restructuring signals making immune cells clean the area in this context. Therefore, SQ at 1*μ*M would promote a M2 profile in macrophages, with the final purpose of cleaning and close the wounds, even if the macrophage is prepolarized at M1 state.

### 3.3. TNF*α* Production

**Figure 3:**
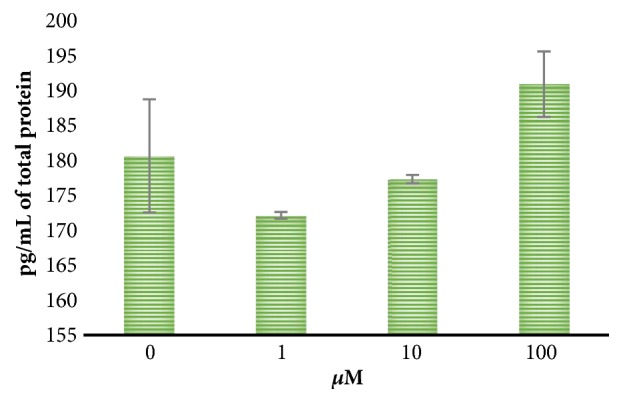
TNF-*α* production after treatment with SQ in M1 macrophages. The results are expressed as pg/mL of the total protein produced. There were no significant differences at p<0.05.

To verify the results obtained by protein-arrays, TNF-*α* was studied using an immunometric assay. Although no significant differences were found, a concentration-dependent increase in TNF-*α* production was observed after treatment with SQ in M1 macrophages and is depicted in Figure 3. This result could be related to the anti- and proinflammatory cytokines studied in early sections. The synthesis of this molecule appeared inhibited at 1 and 10 *μ*M but enhanced at 100 *μ*M. This molecule is one of the main proinflammatory cytokines, activating a proinflammatory response, even the lymphocytes Th1, lymphocytes B, and M1 macrophages recruitment [16].

### 3.4. NF-*κ*B Production

**Figure 4:**
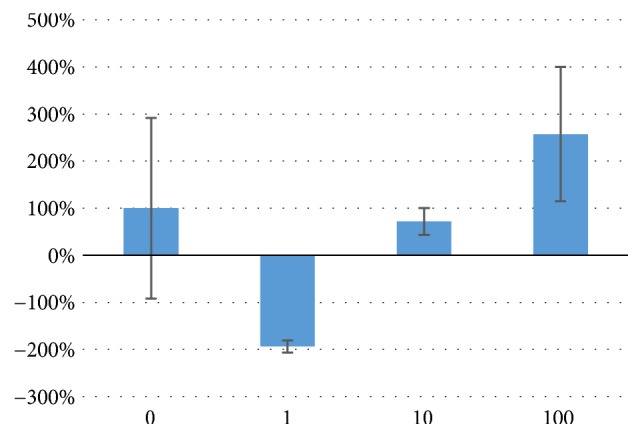
NF-*κ*B production in M1 macrophages after SQ treatment. Data are expressed as mean percentages with respect to the control, which was set at 100%. No significant differences were found at p<0.05.

NF-*κ*B was assayed using ELISA (the results are expressed as percentages with respect to the control). NF-*κ*B production was not altered in a statistically significant manner. Interestingly, in Figure 4, we can observe that, although it is not statistically significant, SQ at 1 *μ*M diminished NF-*κ*B expression. NF-*κβ* is a hallmarked of chronic inflammation and its production is itself regulated, and is precursor of some of the main proinflammatory cytokines which promote the M1 maintenance response [19, 20].

### 3.5. NO Production

The NO production data are expressed as ratios with respect to the control, which was set as 1. In Table 3, we can observe that NO production was not altered by SQ treatments, which, however, decreased at 10 and 100 *μ*M. There were no significant differences between all of the concentrations assayed.

## 4. Discussion

Although there have been few studies that show the role of SQ in inflammation [21, 22], any study describes deeply the action that SQ could have in proinflammatory macrophages, first cells to manage inflammation process in injuries, and its activity related to tissue repair and remodelling inflammation. To simulate an inflammatory process (derived by an infection, neoplasia, or early damage in tissues), we stimulated human monocyte cells to differentiate them into M1 proinflammatory macrophages [5], which are the first immune cells that appear at sites of inflammation. After that, cells were treated with SQ to analyse the macrophage response. Our results show that SQ did not exert any cytotoxic action on macrophages that had a phenotype close to M1 polarization but behaved in two different ways based on its concentration. At a lower concentration (1 *μ*M) SQ mediated an anti-inflammatory response, even though M1 polarization had a proinflammatory profile.

First, SQ appeared to increase the production of IL-10 (Figure 2(a)). IL-10 is an anti-inflammatory cytokine that inhibits antigen presentation and proinflammatory cytokines, clue of several diseases, such as inflammatory bowel syndrome (IBS) [23]. Moreover, IL-4 and IL-13 were enhanced (Figure 2(a)) by SQ (1 *μ*M), both of which, in addition to IL-10, are the main cytokines that promote M2 anti-inflammatory polarization in macrophages [13]. IL-13 and IL-4 are Th2 cytokines that are involved in the anti-inflammatory process and balance the M1 response to manage wound healing process [14, 18].

Second, SQ promotes increases in GMCSF, GCSF, and Eotaxin-2 (Figure 2(b)). These chemoattractants are known to manage eosinophil recruitment, proliferation, and differentiation [24]. Eosinophils are known for their role in asthma and allergies, where they induce tissue damage, a capacity that extends from their traditional role in protecting the host from parasitic worms. However, they are also associated with tissue repair and remodelling [25]. Together with the production of anti-inflammatory cytokines, SQ appears to mediate tissue remodelling and repair through the recruitment of immune cells and production of anti-inflammatory signals. Additionally, TIMP-2 production is enhanced (Figure 2(b)). This is a natural inhibitor of MMP-2 (metalloproteinase-2). MMP-2 is associated with pathological tissue destruction in chronic diseases, such as cancer and arthritis [26]. Therefore, the increased expression of TIMP-2 after SQ treatment would inhibit the protumoral and proinflammatory activities of MMP-2, associated with tissue destruction.

Furthermore, typically hallmarks of chronic inflammation appeared decreased in proinflammatory M1 macrophages, which in turn will be a paradox* per se*. NF-*κ*B production results are shown in Figure 4; at 1 *μ*M, SQ decreased NF-*κ*B production. This molecule has been described to manage proinflammatory responses and promote chronic inflammation [19, 20]. NF-*κ*B regulates itself and the inflammation process induced by other molecules, such as TNF-*α*, which also promotes inflammation [16]. Interestingly, although they were not statistically significant, we observed that the TNF-*α* levels appeared to decrease at 1 *μ*M (Figure 3), which supports the idea of SQ being a natural anti-inflammatory compound that could aid in chronic inflammatory diseases, such as IBS. NF-*κ*B is downregulated during the release of anti-inflammatory cytokines IL-4, IL-13, and IL-10 [27], all of which were increased by SQ treatment, as seen in Figure 2(a). Furthermore, NO production, a signal of chronic inflammation of M1 phenotype macrophages [13, 17], was not affected significantly by SQ treatment but appeared to decrease after SQ treatments (Table 3). As a result, SQ could promote an anti-inflammatory state in these macrophages, which is closer to M2 than M1 phenotype.

On the other hand, at higher concentrations, SQ is not a mediator of anti-inflammatory responses as it is at lower concentrations. At 10 and 100 *μ*M, it lost its anti-inflammatory activity and promoted a proinflammatory response (observed at 100 *μ*M). Indeed, Cho et al. [11] described the antiaging effect that consumption of squalene had, but they pointed out that “the risk-benefit ratio of high-dose squalene supplementation is too high to recommend it for treating skin ageing.” The present work is a probe of the dual activity that the same compound could have, depending exclusively on the concentration of compound used. As long as the quantity is important, the biomolecular processes that are involved in the action of this compound at low levels can change with an increment of the compound. The biomechanism involved in squalene action needs to be deeply studied, but it is reasonable to think that high amounts of this compound could disrupt anti-inflammatory mechanisms, promoting the opposite response, maybe due to an exacerbated reaction to high levels of squalene. Therefore, special attention has to be paid to the concentrations used in future studies because this compound clearly has two different functions depending on the quantity used.

Taking into account that, among others, M1 macrophages produce anti-inflammatory cytokines after SQ treatment, this compound could mediate the M1 response by promoting a switch from M1 into M2 macrophages. This switch has been previously described in tumour development, where molecular signals can switch macrophages between both states [28]. Further studies are needed to confirm this hypothesis; however, SQ could mediate the polarization of macrophages at sites of inflammation.

SQ treatments were not able to reduce proinflammatory cytokines such as IL-1*α* and IL-1*β*, both of which are precursors of IL-8 enhanced (Figure 2(c)). All of these cytokines are responsible for the recruitment of neutrophils that are capable of phagocytosing other apoptotic cells or evacuating the rest of the lesions produced in acute inflammation [29]. If IL-1 and IL-8 promote an inflammatory response, we believe that INF-*γ* would be consequently increased because it is a hallmark of a proinflammatory response [13], which could turn into chronic inflammation and, consequently, into cancer [16], as in ovarian cancer, where INF-*γ* promotes cancer growth [30]. Interestingly, IFN-*γ* was not enhanced by SQ treatments (Figure 2(c)).

Therefore, we can observe that SQ promotes remodelling and tissue repair response signals together with recruitment molecules of neutrophils, responsible for cleaning the area of death cells and detritus. This compound could manage M1/M2 balance to allow the correct wound healing closure.

As a result, SQ appears to manage the inflammatory responses involving wound healing to promote the end of inflammation and the consequent tissue repair and remodelling through pro- and anti-inflammatory signals, but without M1 response, avoiding the maintenance of chronic inflammation. SQ is, maybe, one of the many compounds responsible for the anti-inflammatory effects observed in VOO, as well as its wound healing action.

## 5. Conclusions

In conclusion, SQ could be a useful natural product that can manage wound healing by its immunomodulation of macrophages; the main innate cells involve in wound healing. It could be useful in the last stages of wound healing resolution, due to its anti-inflammatory properties in the last stage of remodelling and wound closure.

## Figures and Tables

**Table 1:** Representation of all the cytokines studied in RayBio human cytokine antibody array.

Cytokine	General function
*Eotaxin*	Eosinophil chemotactic protein

*Eotaxin-2*	Eosinophil chemotactic protein 2

*IL-1α*	Proinflammatory

*IL-1β*	Proinflammatory

*IL-2*	Involved in Treg and Tc regulation

*IL-3*	Proinflammatory

*IL-4*	Antiinflammatory

*IL-6*	Pro- and antiinflammatory

*IL-7*	Lymphocyte maturation

*IL-8*	Recruitment of eosinophils

*IL-10*	Anti-inflammatory

*IL-11*	Proinflammatory

*IL-12 *	Inductor of Th1 response
*p40*	

*IL-12*	Inductor of differentiation of T cells
* p70*	

*IL-13*	Anti-inflammatory

*INF-γ*	Pro-inflammatory

*GCSF*	Granulocyte colony-stimulating factor

*GMCSF*	Granulocyte-macrophage colony-stimulating factor

*I - 309*	Pro-inflammatory

*TIMP-2*	Inhibitor of metalloproteinase 2 (MMP2)

**Table 2:** Cytotoxic effects of SQ on M1 macrophages were represented as the percentage of cell survival. Significant differences were not found at p<0.05.

Concentration of SQ	0 *μ*M	3.12 *μ*M	6.25 *μ*M	12.5 *μ*M	25 *μ*M	50 *μ*M	100 *μ*M
% Cell survival ± SEM	100 ± 7,61	86.3 ± 6,8	87,4 ± 6,7	86,6 ± 7,3	85,1 ± 4,9	97,4 ± 4,2	104,6 ± 1,7

**Table 3:** NO production after treatment with SQ in M1 macrophages. Values are presented with respect to the control. No significant differences were found at p<0.05.

Concentration of SQ	0 *μ*M	1 *μ*M	10 *μ*M	100 *μ*M
production ± SEM	1 ± 0.10	0.96 ± 0.11	0.65 ± 0.18	0.69 ± 0.14

## Data Availability

The data used to support the findings of this study are available from the corresponding author upon request.
